# Oral Surgery Clinic

**Published:** 1890-03

**Authors:** John S. Marshall


### ORAL SURGERY CLINIC.[1]

SERVICE OF PROFESSOR JOHN S. MARSHALL, M.D.

THE patient before you, gentlemen, is a railroad engineer, thirty years of age, who has recently been in a collision, and came out rather badly battered up. He is suffering from a broken wrist, contusions and lacerations about the head and face, and a compound comminuted fracture of the lower jaw. The accident occurred about two weeks ago. The broken wrist, contusions and lacerations about the head and face have been cared for; he is now referred to us for treatment of the fractured lower jaw. The broken bone has been treated since the injury by the ordinary occipito-mental bandage and wiring the teeth, but these fail to hold the ends of the fractured bone in apposition.

Let us now proceed to a critical examination of the mouth and the fractured jaw. First, we notice the crowns of the superior, central, and lateral incisors are fractured obliquely outward and upward, the centrals losing about one-half and the laterals about one-third of their crowns. This indicates a blow upon the lower jaw, near the chin, the direction of the force being upward. The lower jaw is fractured through the alveolus of the right lateral incisor (this tooth is missing, having been knocked out in the accident), and then extends obliquely backward and downward through the body of the bone. The six teeth to the left of this,—viz., three incisors, the cuspid, and the first and second bicuspids, together with their alveolar process, are broken loose from the body of the bone, so that they are freely movable in all directions. The gum is intact along the line of this horizontal fracture upon the labial surface, but upon the lingual surface it is considerably lacerated, and the bone is here, and at the ends of the fracture, exposed to the secretions of the mouth. Suppuration has taken place, as generally happens in compound fractures opening into the mouth, owing to the fact of the great difficulty in maintaining an aseptic condition of the parts.

Until the introduction of the antiseptic method of treating wounds, compound fractures, in any location of the body, were

---

[1] Delivered before the students of the Medical and Dental Departments of the Northwestern University, at St. Luke's Free Hospital, Chicago, Ill.

generally followed by suppuration. Vigorous efforts should be made, however, to combat these conditions by the free use of antiseptic mouth-washes, and if this treatment is faithfully carried out, in many cases it will prove successful. The mouths of the most cleanly persons are the habitat of great numbers and various forms of micro-organisms, while the food and the fluids of the mouth, loaded with these organisms, cannot be prevented from entering the wounds; consequently, fermentative processes are set up, and pathogenic bacteria readily find access to the tissues.

This fracture is a somewhat peculiar one, and may result in loss, from necrosis, of that portion of the alveolar process, and the teeth contained in it, which are broken loose from the body of the jaw. Injuries to the bones of the face, however, are less likely to result in necrosis than like injuries in other portions of the body, which is due to the greater vascularity of the tissues of the face. You will notice that the teeth occlude only upon the right side, posterior to the line of fracture, through the body of the bone, the space between the upper and lower teeth of the left side is fully three-eighths of an inch, while the movable fragment is carried inward and to the right fully one-half of an inch.

In order to treat this case successfully, it will be necessary to construct an interdental splint, with metal arms passing out at the corners of the mouth, and turned backward on a line with the lower jaw, and outside the cheeks. This form of splint is known as the Kingsley.

After plaster casts have been made from impressions taken, we saw through the lower one upon a line with the fractures, and adjust the fragments of the cast to their normal position by the aid of the cast of the upper jaw. If the occlusion of the teeth of the casts is made properly, and the fragments of the cast of the lower jaw united with plaster of Paris, in their new position it will represent the shape and position of the jaw before the fractures occurred; and a splint constructed to fit this will bring the fragments of bone into their normal position. It is made secure by placing a compress under the chin at the most depressed portion of the jaw, and passing a bandage back and forth over the arms of the splint and under the chin.

[The splint was constructed of hard rubber with soft steel arms, and inserted two days later. At the end of two months, union of the bone had taken place in each of the fractures and the occlusion was perfect.]

